# Use of Polyamide (Nylon) Cable Ties for Vascular Ligation of Healthy Equine Jejunal Mesentery

**DOI:** 10.3389/fvets.2021.639424

**Published:** 2021-08-12

**Authors:** Samuel D. Hurcombe, Holly A. Roessner, Chelsea E. Klein, Julie B. Engiles, Klaus Hopster

**Affiliations:** ^1^Department of Clinical Sciences, School of Veterinary Medicine, New Bolton Center, University of Pennsylvania, Kennett Square, PA, United States; ^2^Department of Pathobiology, School of Veterinary Medicine, New Bolton Center, University of Pennsylvania, Kennett Square, PA, United States

**Keywords:** mesentery, ligation, equine, jejunum, cable tie

## Abstract

Jejunal vascular ligation is an essential step in performing jejunojejunostomy. Hand sewn ligation is typically used and can increase operative time with long sections of bowel to be removed. Nylon cable ties (NCT) have been used for vascular ligation in horses but are yet to be investigated for application on the mesenteric vasculature of the gastrointestinal tract. Our objective was to evaluate the efficacy and short-term safety of NCT jejunal mesenteric vessel ligation in healthy horses. Eight healthy adult horses underwent midline celiotomy. A segment of jejunal mesentery was identified (≥4 arcades). Briefly, three fenestrations (proximal, middle, distal) were made 5–10 mm apart adjacent to the first and last vascular arcade to be ligated. Two sterilized NCT were passed to encircle the mesentery through the proximal and middle fenestrations, separated by intact mesentery. NCT were closed tightly and the vascular pedicle transected with Mayo scissors through the distal fenestration. Jejunojejunostomy was then performed and the mesentery sutured closed. The number of vascular arcades and time to ligate using NCT were recorded. At 2 weeks, horses underwent repeat celiotomy to assess the healing of the NCT ligation site and an equal number of vascular arcades were hand sewn double ligated using 2-0 Polyglactin 910 as a timed comparison. NCT mesenteric ligation was significantly faster than hand sewn methods (*P* < 0.01). Effective hemostasis was achieved in all cases. There was no evidence of local infection or adhesions at 14 days post-operatively. Further investigation in the long-term effects in horses as well as horses with strangulating jejunal lesions are needed for clinical application.

## Introduction

Small intestinal disease represents about 25–64% of colic cases where 58–85% have a strangulating lesion. Resection and anastomosis (R&A) is frequently required to correct the lesions representing a significant amount of the total anesthetic time (1). Ligation of the vascular arcades associated with segments of bowel to be resected is an essential initial step in performing R&A of the bowel. Extensive small intestinal R&A can require transecting many arteries which takes considerable time. Prolonged surgery (and anesthesia) times have been associated with decreased survival (2). In existing literature, there are limited data describing mesenteric vascular ligation methods in horses (3, 4). Use of hemostatic clips, electrothermal coagulation and sutured ligations are considered mainstays for achieving mesenteric vascular occlusion and hemostasis as described in large animal surgery texts (1, 5). Disadvantages of handsewn ligation can be slippage, hemorrhage and increased operative time. Disadvantages of electrothermal coagulation include expense of the equipment and limited vessel size (≤7 mm) sealing ability (3). Recently, the LDS (Autosure LDS 15 W; Ligate Divide and Staple) a carbon dioxide driven automated device became unavailable to purchase and use as the manufacturer discontinued its production. The LDS was an attractive alternative to hand sewn ligations in colic surgery. The LDS reliably discharged two “C” shaped hemostatic clips and cut between the two to achieve fast mesenteric vessel ligation. In the absence of the LDS, surgeons may use hemostatic clips (Ligaclip®, Ethicon Endosurgery LLC), an electrothermal coagulation and fusion (Ligasure®) device and/or perform two or three encircling suture ligations per arcade to achieve hemostasis. As such, we believe there is a need to find an alternative method for vessel ligation that is safe, inexpensive and efficient.

Cable ties (CT) have been used in veterinary medicine for a variety of applications including neonatal foal rib stabilization (6, 7), ovariohysterectomy in cats (8), splenectomy in cats (9) and rhesus monkey (10) and nephrectomy in pigs (11). In horses, CT have been used for ovarian vascular pedicle ligation in mares (12), rescue ligation during splenectomy (13), cecocolic intussusception correction (14) as well as colonic vascular ligation in preparation for colonic R&A (SDH personal observation).

We believe that nylon CT (NCT) may be a useful alternative to handsewn ligation for fast and effective mesenteric vessel ligation in horses undergoing jejunal R&A. Based on previous studies in animals, there is both experimental and clinical support for the use of a cable tie construct for vessel occlusion in colic surgery in horses.

Our objectives were to describe an effective, inexpensive and time efficient technique for mesenteric vessel ligation in horses undergoing jejunojejunostomy. We hypothesize that NCT ligation will be a fast and effective method of jejunal mesenteric vessel ligation compared to handsewn jejunal mesenteric vessel ligation.

## Materials and Methods

### Study Design

This study was approved by the institutional animal care and use committee (protocol # 806545) at The University of Pennsylvania. *A priori* sample size calculation was based on pilot data of ligation times between handsewn (single ligature) and thermocoagulation (Ligasure®) methods for five vascular arcades. With a power of 80% and type 1 error of 0.05, we concluded eight horses would be needed.

Eight university owned animals were used in this study. All horses were > 1 year of age without any known or recent history of abdominal disease. Horses were housed in temperature controlled indoor stalls. Food was withheld 12 h before surgery and water withheld 6 h before surgery.

### Procedure(s)

#### Preoperative Preparation and Anesthesia

A 14-gauge catheter was aseptically placed in the left jugular vein and horses were administered procaine penicillin G (22 mg/kg IM), gentamicin sulfate (6.6 mg/kg IV) and flunixin meglumine (1.1 mg/kg IV). Horses were premedicated with acepromazine (0.05 mg/kg IV), xylazine (0.8 mg/kg IV) and induced with midazolam (0.05 mg/kg IV) and ketamine (2.2 mg/kg IV). Anesthesia was maintained using desflurane and oxygen with and end-tidal inhalant concentration targeted of 8 Vol.%. Intravenous balanced isotonic crystalloid solution was administered (5 ml/kg/h) for the duration of the procedure. All horses were instrumented for invasive blood pressure monitoring and cardiac output monitoring by thermodilution using a Swann-Ganz catheter. Pressure support was achieved with dobutamine as a continuous rate infusion as needed to maintain a mean arterial blood pressure (MAP) above 70 mmHg.

#### Surgical Procedure

All surgical procedures were performed by the same surgeon (SDH). Horses were aseptically prepared and draped for a ventral midline celiotomy. A standard 30 cm incision was made into the abdomen. A brief exploration of the abdomen was performed to ensure no lesion or abnormality was encountered.

Next, the small intestine was exteriorized and lumen contents were decompressed into the cecum from oral to aboral. A section of mid-distal jejunum was identified for proposed vascular ligation to include at least four but up to 12 vascular arcades, corresponding to “at minimum” 150 cm (5 feet) of jejunum. The number of arcades ligated was randomized using a number generator (www.random.org) with range limits of 4 and 12. The middle to distal jejunal site was selected for its longer mesentery compared to proximal segments, aiding access to perform rescue ligation if NCT ligation failed (i.e., ineffective hemostasis, slippage, NCT failure to lock).

##### NCT Specifications

8 mm cable width, 45.72 cm cable length, 13 mm total locking head width, 79.4 kg loop tensile strength, −40°F (−40°C) to 185°F (85°C) stability, and clear-white (non-colored). (Cable ties, product number 0292679, Utilitech, L G Sourcing Distribution, N Wilkesboro, NC, USA). NCT were packaged in pairs and sterilized using ethylene oxide (EO) gas.

#### Application of the NCT

The mesentery was fanned out to visualize the proposed vessels to be ligated. Metzenbaum scissors were used to make three 5–8 mm fenestrations [proximal (closest to the mesenteric root), middle and distal (closest to the jejunum)] ~5–10 mm apart on the aboral aspect of the distal vessel to be ligated and the orad aspect of the proximal vessel to be ligated such that when encircled, both vessels and all vessels between would be incorporated (Supplementary File 1). It is of notable importance to ensure the fenestrations are separated by intact mesentery to minimize potential NCT slippage. We attempted to make the length of the fenestrations less than the cable width purposefully. A smaller (~5 mm) fenestration was able to accommodate the 8 mm cable width without additional tearing in healthy mesenteric tissue. The distance from the bowel to the distal fenestration was approximately 15 cm.

The first NCT was placed through the proximal fenestration of the aborad side around the jejunal mesentery to be resected and exited out the proximal fenestration of the orad side. The second NCT was placed in the middle fenestration in similar fashion (Supplementary File 2).

With an assistant helping to straighten out the mesentery, the first NCT was closed down as tightly as possible by hand strength followed by the second NCT. Each was checked twice to ensure as closed position as possible around the mesentery and to ensure no mesentery was entrapped in the locking box of the NCTs. When closed, the mesentery between the two NCT was observed to bulge. A curved Ochsner forcep was then placed in the distal fenestration to clamp the mesentery distal to the second NCT, closest to jejunum. Curved Mayo scissors were then used to cut the mesentery from the distal fenestrations close to the Ochsner forcep (Supplementary File 3).

The resultant mesenteric stump was inspected for active hemorrhage. If no hemorrhage was observed, the tail lengths of the NCT were cut using Mayo scissors 2–3 mm close to the locking box of the NCT. A rescue procedure was preplanned for if excessive hemorrhage from the transected stump occurred. This involved having enough Kelly hemostats for at least equal to the number of arcade vessels being ligated. The vessels would then be subsequently double ligated using 2-0 Polyglactin 910 suture.

Finally, to complete vascular ligation in preparation for end-to-end jejunojejunostomy, vessels closest to the proposed jejunum to be transected were hand ligated using two encircling ligatures with a surgeons' knot and four additional throws using 2-0 Polyglactin 910. The total number of vascular arcades ligated, the time to perform complete jejunal arcuate vessel ligation, total length of bowel resected, presence/absence of complications associated with NCT application were all recorded. The time to ligate per arcade was calculated by the total ligation time/number of arcades ligated.

Following an end-to-end jejunojejunostomy (separate study comparing handsewn 1- or 2-layer inverting patterns using 2-0 polydioxanone), the mesenteric defect was closed by suturing the mesentery to the free end of the mesenteric stump created by vascular ligation (**Figure 2A**) followed by a simple continuous line to the mesenteric attachment of the jejunum using 2-0 Polyglactin 910 (Supplementary File 4). Carboxymethylcellulose (1 L) prepared and sterilized in the hospital pharmacy, was instilled into the abdomen to help prevent adhesion formation following jejunojejunostomy. The abdominal wall was closed in three layers and horses were recovered with head and tail rope assistance.

#### Post-operative Care

Post-operatively, horses were monitored by physical examination every 6 h for 3 days then every 12 h for 11 days. PCV/TP was assessed every 12 h for 3 days. Horses were treated with antibiotics (procaine penicillin G 22 mg/kg, IM, every 12 h and gentamicin sulfate 6.6 mg/kg, IV, every 24 h; 3 days), flunixin meglumine (1.1 mg/kg, IV, every 12 h; 3 days) and monitored in the intensive care unit for signs of colic, internal hemorrhage and other postoperative morbidity. Horses were gradually refed over the initial 3–5 days from surgery and housed for a total of 2 weeks.

#### Re-Laparotomy

Horses were fasted 8 h prior to a second laparotomy before being anesthetized in similar fashion to the first laparotomy as part of the separate jejunojejunostomy study. The jejunal NCT site was inspected for presence/absence of adhesions, hyperemia, hematoma formation, or infection. The site was harvested and placed in 10% buffered formalin for histopathological assessment. Following completion of a separate ventilation study, horses were humanely euthanized under general anesthesia using potassium chloride (1.5 mmol/kg IV bolus) as accepted by the American Veterinary Medical Association. Confirmation of death was by asystole noted on electrocardiography and lack of auscultable cardiac sounds.

#### Sutured Ligation Times

A comparison of hand sewn ligation times was then performed by using 2-0 Polyglactin 910 and two encircling ligatures (surgeons knot with 4 additional throws/ligation). The number of arcades ligated was equal to the number of jejunal vessels ligated using NCT for a direct timed comparison of techniques. The additional smaller vessel ligations performed to complete the jejunojejunostomy at the first surgery were not included in the NCT ligation time recorded and were not performed in the hand sewn timed comparison.

#### Pathology

A single board-certified pathologist (JBE) performed all gross and histopathology evaluations. Jejunal mesentectomy sites were grossly evaluated for the presence of external adhesions, granulation tissue or fibrinous exudates (Table 1). Tissues were serially transected and grossly inspected for internal lesions compatible with inflammation (e.g., fibrin pockets, abscesses or granulomas). Representative sections that included the NCT-tissue interface as well as serosal surface were trimmed and routinely processed for histopathology, including paraffin embedding, 5 μm-thick microtome sectioning and staining with Hematoxylin and Eosin (H and E), Phosphotungstic Acid Hematoxylin (PTAH) for fibrin, and Picrosirius Red (PSR) for fibrosis. Because of the unique anatomic features pertaining to the relative large amount of equine mesenteric tissues incorporated into the NCT device in this study that precluded extrapolation of histologic grading schemes developed for evaluation of ligature devices in other animal models, the authors developed a semi-quantitative histologic scoring system to evaluate the NCT-tissue interface, serosal surface and subserosal mesentery to characterize inflammation, necrosis, fibrin, hemorrhage, granulation tissue, fibrosis and serosal rifts and/or adhesions (Table 2).

**Table 1:** Frequency of assessed morbidity associated with NCT application to Jejunal mesentery.

	**Present**
Hyperemia at NCT site	3/8 (37.5%)
Hematoma at/near NCT site	1/8 (12.5%)
Adhesion at NCT site	0/8 (0%)
Adhesion anywhere in the abdomen	1/8 (12.5%)
Local infection at NCT site (i.e., abscess)	0/8 (0%)
Numbers represent fractions and percentages	

**Table 2:** Histopathologic grading scheme to assess healing and inflammation at the NCT site.

**Score**	**Histologic parameters**								
	**Inflammation at NCT interface**	**Mesenteric Fibrin**	**Mesenteric suppurative inflammation**	**Mesenteric mononuclear cell inflammation (lymphocytes, plasma cells, histiocytes)**	**Mesenteric granulomatous inflammation**	**Mesenteric fat necrosis**	**Subserosal granulation tissue**	**Subserosal hemorrhage**	**Mesothelial defects**
0	None to minimal comprising scattered mono-nuclear cells	None	None	None	None	None	Diffuse fibrous maturation	None	Flattened mesothelium without disruption
1	Mild- thin rim predominant non-suppurative	Focal	Focal to mild scattered	Focal	Focal	Focal	Well organized; capillaries <10% area	Mild and scattered; limited to subserosal tissue	Focal to multifocal defects (<2 mm total area) with fibrin
2	Moderate- thick rim, predominant suppurative	Multifocal or regionally extensive	Multi-focally intense or regionally extensive	Multifocally intense or regionally extensive	Multifocal or regionally extensive	Multifocal or regionally extensive	Moderately well-organized; capillaries 11–50% area	Moderate with pockets <1 mm	Multifocal to regional defects (2–10 mm total area) with fibrin
3	Severe- predominant suppurative extending to serosal surface	Diffuse or extending to serosal surface	Diffuse or presence of large (>1 mm) abscess	Diffuse	Diffuse or extending to serosal surface	Diffuse	Poorly organized; capillaries >50% area	Severe with hematoma >1 mm or protruding beyond serosal surface	Large defects (>10 mm total area) with fibrin

### Statistical Methods

All continuous data were assessed for normality by Kolmgorov-Smirnov testing. Parametric data (age, weight, all timed ligation events (NCT and hand sewn), histologic score) are expressed as mean ± SD and non-parametric data (number of arcades ligated, length of bowel resected, length of mesentery from bowel to NCT) are expressed as median (range). Differences in ligation times (total times and ligation time/arcade) between NCT and hand sewn methods were compared using *t*-tests. The difference in time between ligation methods within horse was calculated by subtracting the total NCT time from the total handsewn time. To assess the predicted time saved per vessel ligated, simple linear regression was performed to develop an equation where X = number of vascular arcades and Y = time saved. To compare the magnitude of time saving within horse as a percentage of NCT vs. hand sewn ligations, the following equations for total time and time/vessel were used:

$$\begin{array}{l} 100-\left(\text{NCT time }\left(\text{total}\right)\text{ Hand sewn time }\left(\text{total}\right)\times 100\right);\text{and}\\ 100-\left(\text{NCT time }\left(\text{per vessel }\right)/\text{Hand sewn time }\left(\text{per vessel }\right)\times 100\right) \end{array}$$

All statistical analyses were performed using commercial software (Microsoft Excel, Redman, WA; Prism vs. 8, GraphPad, LaJolla, CA). Significance was set at P < 0.05.

## Results

All eight adult horses completed the experimental protocol. There were five mares and three geldings. All eight horses were Thoroughbreds with a mean ± SD age of 14 ± 5.8 years. The mean ± SD weight was 515 ± 26 kg. No horse had evidence of prior abdominal surgery.

**Figure 1:**
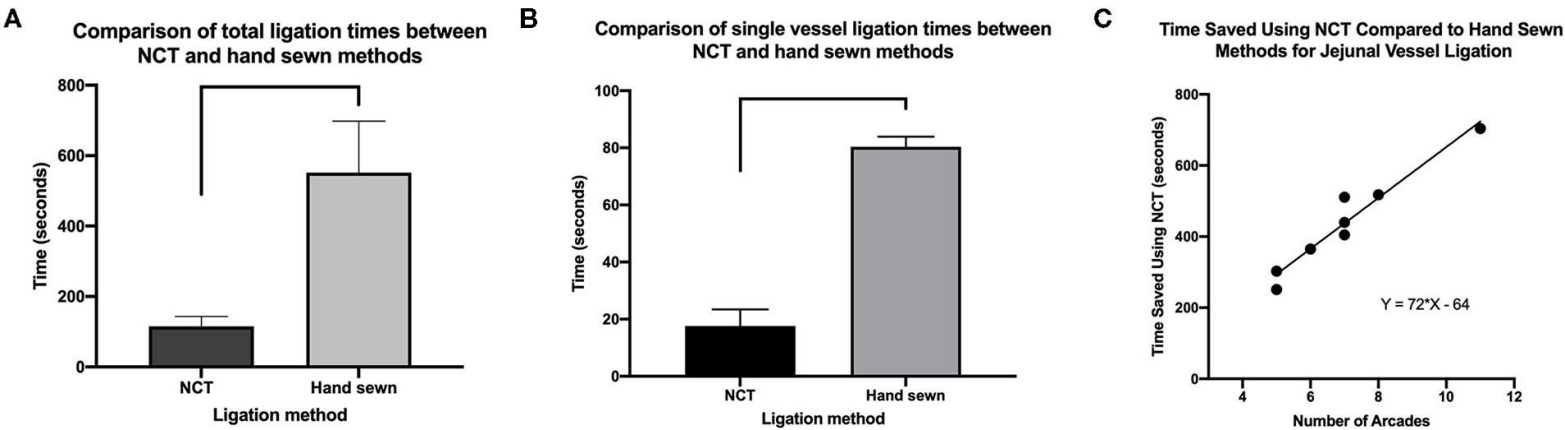
**(A)** Comparison of ligation times (total) **(B)**. Individual (total number of arcades/total time) between NCT and hand sewn methods. Data represent mean ± SD. Significance bars indicate *P* < 0.001. **(C)** Linear regression of time saved per vessel ligated using NCT over hand sewn methods.

The median (range) number of vascular arcades ligated using NCT at the first surgery was 7 (5 to 11). The median length of jejunum resected was 303 cm (240 to 624 cm). The mean total time taken to ligate the jejunal vasculature with NCT was 115 ± 28 s (1.9 ± 0.46 min) vs. 552 ± 146 s (9.2 ± 2.4 min) for hand sewn ligation of an equal number of vascular arcades or on average 78% faster (*P* < 0.001; Figure 1A). Overall, the mean time taken to ligate per vascular arcade was significant faster with NCT (18 ± 5.8 s) compared to hand sewn (80 ± 3.5 s; *P* < 0.001; Figure 1B). On average, NCT was 78% faster than hand sewn methods on a per vessel basis.

The mean total time saved (hand sewn total time minus NCT total time) was 437 ± 142 s (7.3 ± 2.4 min). The time saved per ligation was estimated by the following equation: Y (time saved) = 72 × (number of vessels to be ligated)−64 (constant) with a Goodness of fit = 0.94; *P* < 0.001 (Figure 1C). Based on this equation, a predicted time saving would be found with even a single vessel to be ligated however, the minimum number of vessels ligated in this study was 5 as determined by randomization. The actual time saving for five vessels was 251 s (4 min and 11 s) vs. the predicted time saving for five vessels would be 296 s (4 min and 56 s).

The median distance from the mesenteric attachment to the bowel and the NCT transection site (5 mm distal to the second NCT) was 15 cm (12 to 24 cm). In one horse (horse 7) where 11 arcades were ligated using NCT, we appreciated slight bending of the jejunojejunostomy as a result of differences in the proximal and distal mesentery length. There was no impediment to flow through the anastomosis.

No horse developed hemoabdomen post-operatively based on physical examination and PCV, TP monitoring. Furthermore, no horse developed post-operative ileus following R and A. 3/8 horses developed mild self-limiting colic signs for 24 h immediately post-operatively. A single horse developed a gastric impaction and colic signs at day 5 post-operatively after removing the muzzle and eating straw bedding (horse 1). This was resolved with gastric lavage and gradual refeeding.

Horse 3 had a nephrosplenic entrapment of the ascending colon that was manually corrected prior to the first experimental procedure. This horse did not show any signs of colic preoperatively but did have mild low-grade colic for 24 h post-operatively that resolved without any specific therapy. At the second surgery, there was extensive splenic adhesions to the ventral body wall (midline and left paramedian aspects). There were no other adhesions within the abdomen, notably none associated with the NCT and mesenteric ligation site. An incidental jejunal mesenteric lipoma was found and removed in horse 5. Horse 8 had a hematoma within the mesentery between the NCT site and the mesenteric aspect of the jejunum. The mesentery was smooth, non-reactive and there was no mechanical deformation of the jejunojejunostomy site. The hematoma was largest at the mesenteric aspect of the jejunum perhaps indicating that the hemorrhage arose from a small mesenteric vessel associated with closure of the mesenteric defect rather than from the NCT as there was no evidence of gross hemoabdomen.

Post-operatively, a single horse (horse 5) had an elevated heart rate (44–52 beats per minute) for 48 h. Evaluation of the abdomen for hemoabdomen was performed using transabdominal ultrasound, in addition to PCV and TP assessment and did not reveal any evidence of abdominal bleeding. This horse also showed signs of neck pain following procaine penicillin G injection and so the horse subsequently received a single dose of long acting ceftiofur (6.6 mg/kg) intramuscularly once and was discontinued of additional antibiotics. The heart rate and neck pain resolved without specific therapy.

### Pathology

**Figure 2:**
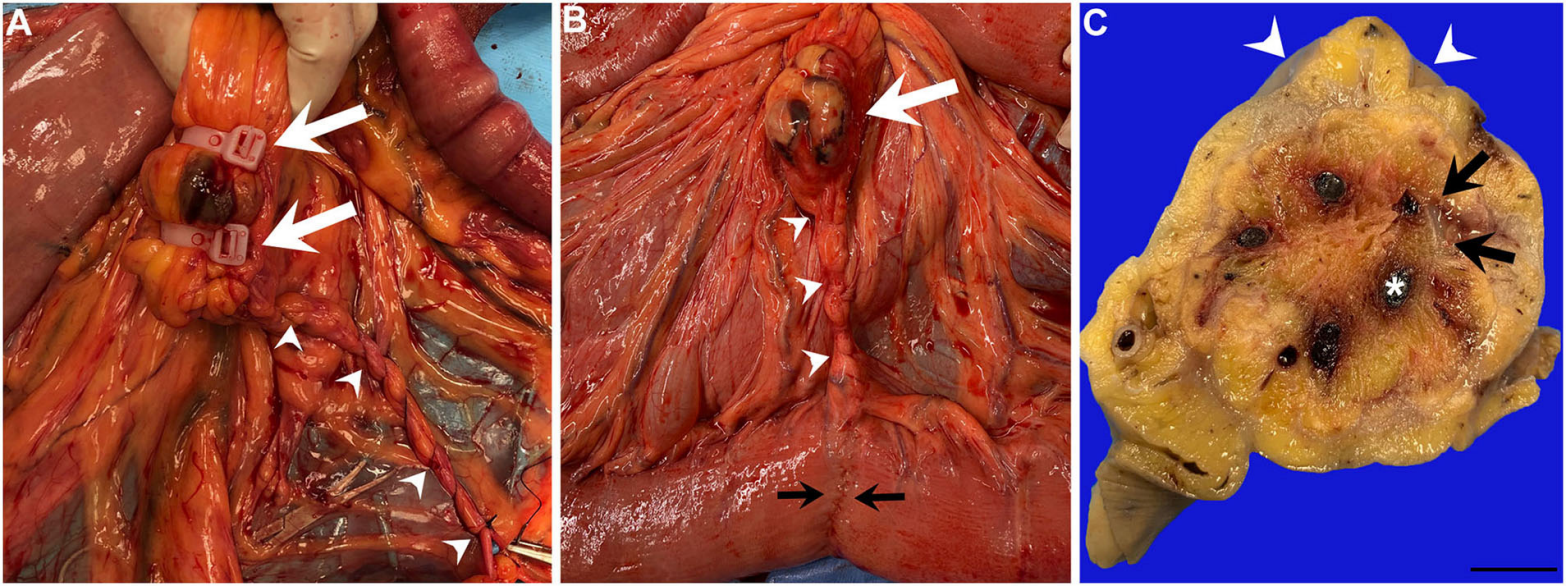
**(A)***In situ* photograph of the jejunal mesenteric NCT placement sites at the time of surgery (white arrows) adjacent to the mesenteric rent site repaired with simple continuous suture pattern (white arrowheads). **(B)** *In situ* photograph of the jejunal mesenteric NCT placement site at the time of the second surgery (white arrow) adjacent to the mesenteric rent repair site (white arrowheads) and jejunojejunostomy site (black arrowheads). **(C)** *Ex situ* formalin fixed serial section of the NCT site shows a portion of the curvilinear NCT cable ring (black arrows) encircling the jejunal vasculature within the center (asterisk); the serosa is encapsulated by smooth white fibrous connective tissue (white arrowheads) (scale bar = 10 mm).

**Figure 3:**
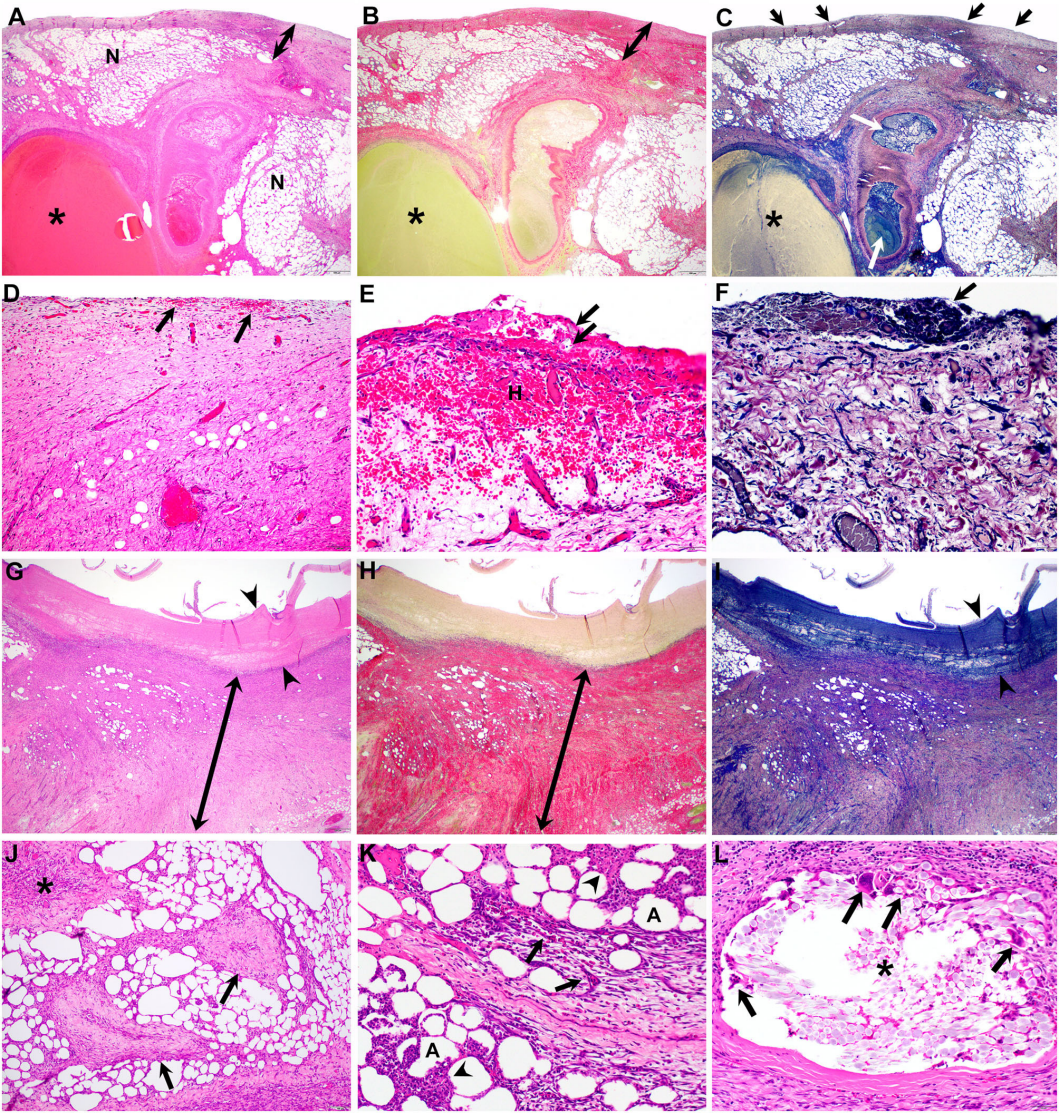
Photomicrographs of H&E (**A,D,E,G, J–L**), PSR (**B,H**) and PTAH (**C,F,I**) stained sections of the jejunal mesenteric remnant at the NCT site. **(A–C)** On low (2×) magnification, the subserosal tissue is expanded by a well-defined rim of collagen-rich granulation tissue (double-headed arrows) that encapsulates islands of necrotic mesenteric adipose (N) with interspersed vasculature including dilated veins (asterisks) and arteries occluded by fibrin aggregates (C, white arrows); tiny accumulations of fibrin are associated with the serosal surface (C-arrows). **(D–F)** High (20×) magnification photomicrographs of the subserosal tissue featured in **(A–C)** reveals small scattered foci of hemorrhage (D-arrows) that rarely coalesce **(E–H)** or extend to the serosal surface, which contains small (~200 μm) aggregates of fibrin (E,F-arrows). **(G–I)** Low (2×) magnification photomicrographs of the NCT-tissue interface shows a thin (<1 mm) rim of fibrin (arrowheads) containing scant to no inflammation surrounded by a thick (>3 mm) rim of organized, collagen-rich granulation tissue (double-headed arrows). **(J–L)** High (10×) magnification photomicrographs of adjacent mesenteric adipose reveal islands of coalescing foci of granulation tissue (J-asterisk) interspersed with sclerotic and stenotic arterioles (J-arrows). Areas of inflammation include small foci of fibrin and necrosis (K-arrowheads) and eosinophilic granulomatous infiltrates with multinucleate giant cells (K-arrows) centered on necrotic adipocytes **(K–A)**. Circumscribing suture material (L-asterisk) used to close the mesenteric rent are eosinophilic and granulomatous infiltrates including numerous and large multinucleate giant cells (L-arrows).

In the majority of horses, gross inspection revealed encasement of the NCT ligation site in mildly hyperemic, smooth fibrous connective tissue without evidence of hematomas, adhesions or infection (Figure 2B). Serial sections confirmed fibrous encapsulation and revealed no gross evidence of inflammation (e.g., pockets of fibrin, abscesses or granulomas) (Figure 2C). On histology (Table 3), NCT ligation sites were encapsulated by a smooth, mostly intact mesothelial lining containing tiny (100–200 μm) surface deposits of fibrin that merged into organized bands of collagen-rich granulation tissue (Figures 3A–F). Central regions of NCT ligation sites comprised islands of necrotic adipose with congested, dilated veins and fibrin-occluded arteries (Figures 3A–F). The tissue interface with the NCT device showed smooth rims of organizing collagen-rich granulation tissue containing few inflammatory cell infiltrates and scant fibrin (Figures 3G–I). The NCT interface was surrounded by islands of necrotic adipose with coalescing foci of organizing granulation tissue that contained stenotic and sclerotic small caliber blood vessels. There was mild to moderate inflammation infiltrating necrotic adipocytes or surrounding suture material associated with the mesenteric closure associated with the jejunojejunostomy (Figures 3J–L).

**Table 3:** Histologic scores for each parameter at NCT omentectomy site for each horse with averages and standard deviation (SD).

**Horse**	**Histologic parameters**								
	**Inflammation at NCT interface**	**Mesenteric Fibrin**	**Mesenteric suppurative inflammation**	**Mesenteric mononuclear cell inflammation (lymphocytes, plasma cells, histiocytes)**	**Mesenteric granulomatous inflammation**	**Mesenteric fat necrosis**	**Subserosal granulation tissue**	**Subserosal hemorrhage**	**Mesothelial defects**
1	0	1	1	1	1	2	1	0	1
2	1	1	1	1	1	2	1	1	1
3	1	1	1	1	1	2	2	1	1
4	0	0	1	1	1	2	1	0	1
5	0	1	0	1	1	2	2	1	1
6	1	1	1	1	1	1	1	1	1
7	0	0	1	1	1	2	1	0	0
8	0	0	0	1	1	2	1	2	0
Mean ± SD	0.4 ± 0.5	0.9 ± 0.6	0.8 ± 0.7	1.0	0.9 ± 0.4	1.8 ± 0.5	1.4 ± 0.5	1.5 ± 0.5	0.6 ± 0.5

## Discussion

We demonstrated that the application of two encircling polyamide (nylon) cable ties around the jejunal mesenteric vessels resulted in rapid ligation and effective hemostasis *in vivo*. While the use of NCT in surgical practice is not new, this is the first report using NCT specifically for jejunal vessel ligation in preparation for jejunojejunostomy in horses.

NCT applied to the gastrointestinal viscera has been described in a single published case report (14). In that case, 4 encircling NCT were placed around an edematous caecocolic intussusception via right ventral colotomy to facilitate intussuscipiens amputation. The horse was discharged alive following successful correction of the lesion and still alive at 10 months following surgery (14). Moreover, the use of NCT on colonic mesentery to facilitate vascular ligation for colon resection and anastomosis has been observed by the first author but published reports are lacking.

Surgery time is an important factor in equine small intestinal surgery with higher odds of non-survival associated with longer surgery times (2). It is therefore paramount to maximize operative efficiency and minimize surgery times where possible to optimize survival of horses undergoing small intestinal surgery. We found that vascular ligation using two NCT in this study significantly reduced the time for mesenteric ligation compared to hand sewn methods. These time saving advantages were predicted to increase with increasing numbers of vessels to be ligated. We recognize that vascular ligation is one part of the surgical procedure and that the absolute time reduction achieved may not significantly affect total surgery time. We submit that any reduction in operating time should be a goal and that NCT ligation offers one possibility for this. Further study is needed to determine what is the maximum number of vessels per pair of NCT used to complete vascular ligation, the potential impact of faster ligation times, and long-term effects of intraabdominal use of NCT on patient outcome.

Another proposed benefit of NCT use is the costs associated with NCT being less expensive than other methods for hemostasis. For example, the cost associated with using NCT for ligation (NCT, packaging and sterilization) is ~$26 USD compared to the cost for using of electrothermal coagulation (Ligasure®), $336 USD, at this institution. Similarly, NCT are less expensive than suture (< $10 USD for 10 NCT). One important difference between these methods, however, is that NCT leaves a permanent implant in the patient. Long-term safety data on the presence of nylon in the abdomen are currently unknown in horses.

Gandini et al., reported on the use of parallel alternating sliding knots (PASK) in mesenteric arteries of the equine jejunum in an *ex vivo* model (4). Compared to a surgeons' knot with four additional throws, the PASK construct was faster to perform (~20% faster) with comparable or higher leaking pressures depending on the suture materials used. In our study, for an equivalent number of vessels, NCT was 78% faster than handsewn ligation and 78% faster on a per vessel basis. Also, while not specifically evaluated, the time to close the resultant mesenteric defect made from performing R and A seemed significantly faster due to all the mesentery being bound and gathered by the NCT. A comparison of mesenteric defect closure time could be pursued to confirm this impression in future studies.

While arterial bursting pressures for a variety of vessel sealing devices are substantially higher than blood pressure experienced *in vivo* (3, 15, 16), an advantage of NCT over sutured ligations is the unlikely possibility of either suture slippage or poor knot security (12, 17). This is due to the unidirectional integrating locking head making it unlikely for the loop to undo once it is made (18). Further, by ensuring an intact segment of mesentery between NCTs, this theoretically minimized the chance of slippage off of the transected mesenteric pedicle. While specific bursting pressures were not measured in this *in vivo* study, no horse experienced hemoabdomen after surgical recovery indicating effective hemostasis.

As reported previously (12), NCT were well-encapsulated in smooth fibrous connective tissue after 2 weeks of healing in the horses reported in this study. The tissue reaction to NCT observed in these mesenteric tissues was less than what is reported for suture material used in equine jejunal resection and anastomosis procedures at 14 days (19–21). Unfortunately, we did not have histopathology of sutured mesenteric vessels as a control. We recognize that sutured mesenteric ligation or an anastomosis using absorbable suture will ultimately result in healed connective tissue and that longer term assessment of the host reaction to NCT within the equine abdomen is needed. In one small animal report of >10,000 bands in dogs and cats, no known adverse reactions were observed in a 13-year time span but no specific follow-up was pursued either (22). However, there are several reports describing NCT-associated complications in dogs years after placement (23–25) including fistula formation (24) and complications associated with granulomatous inflammation and adhesion/scar tissue formation causing local organ dysfunction (25). Long term follow-up (months, years) are needed to determine whether similar tissue reactions occur in horses. Two case reports describe intra-abdominal use of NCT with no adverse reactions noted at follow-up 7 and 10 months, respectively (13, 14). In our horses, at 2 weeks there was no gross or histopathological evidence of adverse inflammation or a foreign body response demonstrated by smooth, organizing, adhesion-free, fibrous encapsulation at the serosal interface and no histologic evidence of giant cell or macrophage influx at the NCT-tissue interface. Interestingly a paucity of inflammation at the NCT interface at 14 days is in contrast to the moderate eosinophilic and granulomatous inflammation associated with the suture material used for repair of the mesenteric rent associated with the jejunojejunostomy procedure as has been previously recognized (26). It is possible that early granulomatous host reactions were present and missed, again further necessitating the need for long term follow-up to determine the safety of NCT use in the equine abdomen.

Nylon suture materials (non-colored) are biologically inert and non-capillary in the monofilament form (27). Nylon products have been manufactured for use in medicine under good manufacturing practice (GMP) standards including suture and mesh implants (e.g., Supramid®, S. Jackson, Inc.) for a variety of applications. These standards guarantee consistency in product quality and safety. An important consideration is that the NCT used here have not been manufactured under GMP standards for medical application. As a family of polymers with a diverse range of uses, nylon may be treated in various ways to ease molding and softening of the materials creating chemical residues that may be present on commercially available NCT. As such, we recommend that at a minimum, NCT should be cleaned and sterilized as with any medical instrument. We sterilized the NCT in this study using ethylene oxide (EO) gas which is recommended to minimize elongation and deformation of the material as well as have minimal effect on ligature strength (28). Until longer term studies evaluating the tissue responses to NCT within the abdomen are known, GMP for manufacturing medical grade NCT are developed or a bioabsorbable CT construct is available, we caution users of non-GMP NCT for the purpose described here to consider the potential for harmful effects beyond the 14-day investigation time of this study.

A bioabsorbable cable tie construct made of polydioxanone has been produced and used for ovarian pedicle ligation in dogs and renal artery ligation in pigs (11). The investigators evaluated the material properties (inherent viscosity, tensile strength (load vs. strain curve), and deformation) and effects *in vivo* (hemostasis, tissue grip/ligature slip off force) and found the device was more than suitable for effective and secure hemostasis under clinical conditions highlighting the usefulness of the cable tie construct (11). Further, Da Mota Costa et al. reported the use of a resorbable polyglycolic based co-polymer (LigaTie®) in a case-control study of mesovarium ligation in dogs. Use of the Maxon® material equivalent cable tie construct was significantly faster than hand ligation with a single encircling suture (*P* = 0.02) (29). We found a similar significant reduction on ligation time using NCT vs. hand sewn ligation in the horses of this study when at least 5 arcades were ligated. We believe this is a significant advantage and might be particularly useful to shorten surgery and anesthesia times in critical patients that can impact survival (2). Further investigation in large animal species using bioabsorbable cable ties for a variety of purposes such as deep abdominal vessel ligation, splenectomy, nephrectomy and mesenteric vessel ligation are needed.

Limitations in the current experimental design include but are not limited to the fact we assessed the efficacy of NCT ligation in healthy animals. Horses with strangulating small intestinal lesions often have edema, hemorrhage and increased friability of the tissues. It is unknown whether NCT ligation would be suitable in the clinical patient, which is a logical next step to assess. The procedure described in this paper allowed for a “rescue procedure” should NCT fail and this should also be adopted to clinical patients. However, we speculate that NCT would be suitable in diseased tissue as described by de Bont in their successful application of NCT to severely edematous cecal tissue whereby the larger surface area of the NCT might provide better resistance than to suture to “cut through” (14).

Strangulating small intestinal lesions often involve the ileum which has a unique blood supply (1, 30). The utility of using NCT for vascular ligation of the jejunum could expedite mesenteric vessel ligation in larger segments of bowel involving the jejunum, however hand sewn ligation or use of a thermocoagulation sealing device may also be required for ileal vessel ligation in horses requiring jejunoileostomy or jejunocecostomy.

We found that the NCT ligation technique did not cause significant mesenteric length disparity and resultant anastomotic kinking. In the horse with 11 arcades ligated, there was slight bending at the anastomosis but this did not result in intestinal distortion or impediment of ingesta flow. We speculate that in horses with longer segments of bowel being resected, there is potential for anastomotic kinking due to greater mesenteric length disparities created using NCT ligation. Further study investigating the optimal location for NCT placement on the mesentery is needed to mitigate creation of overly short mesentery and mesenteric disparity particular for longer segments of bowel.

Another limitation relates to the number of ligations and utility of NCT over handsewn methods. Based on linear regression analysis, lesions requiring fewer numbers of vessels to be ligated [i.e., (1–3)] are unlikely to yield clinically significantly shorter ligation times when using NCT. Furthermore, we cannot conclude than NCT used for 1 or 2 vessels is safe as these data are lacking. As such we caution surgeons to only consider NCT ligation for 5 to 11 vessels based on the data presented here. Further study on NCT ligation of <5 and > 11 vessels are needed to determine safety and time saving benefit.

Finally, there may be variable hand strength when tightening the NCT into the locked position between operators. One consideration is that closure of the NCT is not automated like when using the LDS or similar stapling device. Instead, human hand strength is required to tighten the NCT and occlude vessels within mesenteric tissue. For this reason, two encircling NCT were placed with intact mesenteric tissue between the two to (a) minimize longitudinal slippage off the mesenteric pedicle and (b) have two points of vascular occlusion for safety as the possibility of human error in locking down the NCT all could result in serious failure. It may be possible to use a single NCT with assured safety, but this is yet to be proven.

In conclusion, NCT use in healthy horses resulted in expedient and efficacious mesenteric vascular ligation without evidence of complication at 2 weeks post-surgery. Future studies evaluating bioabsorbable CT constructs, number of NCT used, as well as long term use of NCT in clinical cases of jejunal strangulation are needed to assess the utility of CT in equine abdominal surgery.

## Data Availability Statement

The raw data supporting the conclusions of this article will be made available by the authors, without undue reservation.

## Ethics Statement

The animal study was reviewed and approved by The University of Pennsylvania Protocol number 806545.

## Author Contributions

SH was involved with experimental design, surgical procedures, data collection, statistical analysis, and manuscript preparation. HR was involved in surgical procedures, data collection, and manuscript review. CK was involved in surgical procedures, data collection, and manuscript review. JE was involved in pathology processing and analysis and manuscript preparation. KH was involved with anesthetic procedures, data collection, and manuscript review. All authors contributed to manuscript revision, read, and approve the submitted version.

## Conflict of Interest

The authors declare that the research was conducted in the absence of any commercial or financial relationships that could be construed as a potential conflict of interest.

## Publisher's Note

All claims expressed in this article are solely those of the authors and do not necessarily represent those of their affiliated organizations, or those of the publisher, the editors and the reviewers. Any product that may be evaluated in this article, or claim that may be made by its manufacturer, is not guaranteed or endorsed by the publisher.

## Supplementary Material

Supplementary File 1 Creating mesenteric fenestrations for NCT placement.

Supplementary File 2 Placement of two NCT around the jejunal vasculature.

Supplementary File 3 Placement of Ochsner forcep and transection of the jejunal vasculature.

Supplementary File 4 Closure of the mesentery.
